# Mesenchymal stem cell-based bioengineered constructs: foreign body response, cross-talk with macrophages and impact of biomaterial design strategies for pelvic floor disorders

**DOI:** 10.1098/rsfs.2018.0089

**Published:** 2019-06-14

**Authors:** Shayanti Mukherjee, Saeedeh Darzi, Kallyanashis Paul, Jerome A. Werkmeister, Caroline E. Gargett

**Affiliations:** 1 The Ritchie Centre, Hudson Institute of Medical Research, Clayton, Victoria 3168, Australia; 2 Department of Obstetrics and Gynaecology, Monash University, Clayton, Victoria 3168, Australia; 3 CSIRO Manufacturing, Clayton, Victoria 3168, Australia

**Keywords:** mesenchymal stem cells, pelvic organ prolapse, macrophages, M1, M2, foreign body reaction, tissue engineering, biomaterials, immunomodulation

## Abstract

An excessive foreign body response (FBR) has contributed to the adverse events associated with polypropylene mesh usage for augmenting pelvic organ prolapse surgery. Consequently, current biomaterial research considers the critical role of the FBR and now focuses on developing better biocompatible biomaterials rather than using inert implants to improve the clinical outcomes of their use. Tissue engineering approaches using mesenchymal stem cells (MSCs) have improved outcomes over traditional implants in other biological systems through their interaction with macrophages, the main cellular player in the FBR. The unique angiogenic, immunomodulatory and regenerative properties of MSCs have a direct impact on the FBR following biomaterial implantation. In this review, we focus on key aspects of the FBR to tissue-engineered MSC-based implants for supporting pelvic organs and beyond. We also discuss the immunomodulatory effects of the recently discovered endometrial MSCs on the macrophage response to new biomaterials designed for use in pelvic floor reconstructive surgery. We conclude with a focus on considerations in biomaterial design that take into account the FBR and will likely influence the development of the next generation of biomaterials for gynaecological applications.

## 1. Introduction

Pelvic organ prolapse (POP) is a common debilitating condition affecting 25% of all women. POP is the herniation of pelvic organs into the vagina with symptoms of bladder, bowel and sexual dysfunction [1]. Although vaginal childbirth is the main risk factor, the POP aetiology is multi-factorial; ageing, obesity, pregnancy, parity, genetics, history of diabetes and hypertension impact its progression [2]. Prevalence of POP varies in different geographical regions. The annual POP incidence in the USA is reported to be 31.8% over 2–8 years in a follow-up study in menopausal women [3]. The rate of vault prolapse is reported to be between 4.4% and 6–8% in two European countries, Italy and Austria, respectively [4,5] and the mean prevalence in developing countries is about 19.7% [6].

Surgical and non-surgical or conservative therapies are currently offered for POP treatment and patient preference is important in the type of treatment chosen. Conservative methods include pessary and pelvic floor muscle training (PFMT). Pessaries are ring-shape plastic or silicone materials, inserted into the vagina and provide support for the affected pelvic organs in women with early stages of POP [7]. While they can help alleviate some of the symptoms associated with POP, they do not assist in the repair of the damaged vaginal tissues [8–11]. PFMT is mostly offered by physiotherapists with expertise in women's health. Pelvic floor exercises improve muscle function which ultimately increases pelvic floor strength. There is some available evidence showing the positive effect of PFMT on POP symptoms including quality of life and prolapse severity; however, its long-term effectiveness still needs investigation [12,13].

Reconstructive native tissue surgery is offered based on the severity of POP and patient preference. Reconstructive surgery will correct the prolapsed vagina and maintain or improve sexual function while relieving pelvic symptoms. Unfortunately, data from studies carried out between 1995 and 2017 revealed an average recurrence of 36% following native tissue reconstructive surgery [14].

Owing to the relatively high failure rate of surgery, POP treatment often used synthetic polypropylene meshes in transvaginal pelvic floor reconstructive surgery. However, in approximately 10% of women, vaginal meshes led to serious adverse events associated with serious foreign body reactions (FBR) including mesh exposure and erosion. Following several FDA warnings [15,16] market withdrawal of most products [17,18], litigation and banning of pelvic meshes in several jurisdictions, there is no definitive cure for POP [19].

Current research is now focused on improving biocompatibility and interactive tissue properties of mesh rather than using inert implants [20], including the use of adult stem cells from several sources [21–23].

Impressive advances in biomaterial design and tissue engineering have demonstrated that proactive control of host cell responses may be beneficial and improve implant performance [24]. Cellular responses at the implantation site trigger an FBR that ultimately determines rejection or integration of the implanted biomaterial. It is pivotal that the new generation of pelvic floor biomaterials actively interact with tissue rather than merely have an inert presence, to promote healing and integration. To this end, tissue engineering approaches have employed structural and cellular cues for the optimal reconstruction of damaged tissues [22,25,26].

Tissue engineering combines biomaterial scaffolds, therapeutic cells such as mesenchymal stem cells (MSCs) or immunomodulatory or anti-inflammatory factors to achieve tissue repair [27]. An increasing body of evidence points to the benefits of such approaches over traditional inert implants [28]. The immunomodulatory properties of MSCs have been exploited in numerous clinical trials for chronic inflammatory disorders, including graft versus host disease and autoimmune diseases. Recent studies have also shown the benefits of cell-based tissue engineering in reproductive health [22,29,30] including POP, and ovarian regenerative medicine [31].

Regardless of the implantation organ, cell-based or cell-free bioengineered surgical constructs will likely provoke an inflammatory macrophage-associated FBR. A growing body of evidence highlights the need for a deeper understanding of the interactions between immune cells and surgical implants as these dictate the FBR response, which ultimately determine success or failure of implanted pelvic support constructs. It is therefore imperative to not only improve design and materials but to also understand as well as control the FBR they trigger for overcoming current mesh-related challenges such as erosion and pain. Development of tissue engineered constructs containing immunomodulatory cells such as MSCs impact several cell types and pathways of the immune system that modulate deleterious FBR responses. A detailed knowledge of the mechanisms involved is indispensable to ensure appropriate integration of tissue engineering constructs in host tissues. FBR profiling is critical to determine the long-term efficacy of all medical devices, and circumventing such studies generating this knowledge may disrupt clinical practice [32] as exemplified by the rise and fall of pelvic mesh usage [20]. This review will focus on key aspects of the FBR to bioengineered MSC-based implants for women's health, particularly application to pelvic floor disorders. We discuss the key macrophage players, their immunobiology, their cross-talk with MSCs and the impact of biomaterial design on the FBR. We also discuss the immunomodulatory effects of endometrial mesenchymal stem cells (eMSCs) on the macrophage response to new biomaterials and their potential for gynaecological applications [33,34].

## 2. Macrophages: origin, function and plasticity

### 2.1. Origin

Monocytes, macrophages and dendritic cells are phagocytic cells originating from myeloid precursors in the bone marrow [35]. Macrophages are distributed in most tissues in varying numbers and contribute to tissue haemostasis by responding to foreign materials and producing an array of bioactive molecules [36]. It was previously believed that macrophages are only derived from circulating monocytes [37]. Previously, definitive fate mapping studies revealed that tissue macrophages of some organs (lung, liver and spleen) are generated during embryonic development and maintain themselves by self-renewal during adulthood, rather than replenishment by circulating monocytes [38].

### 2.2. Function and plasticity

**Figure 1.**
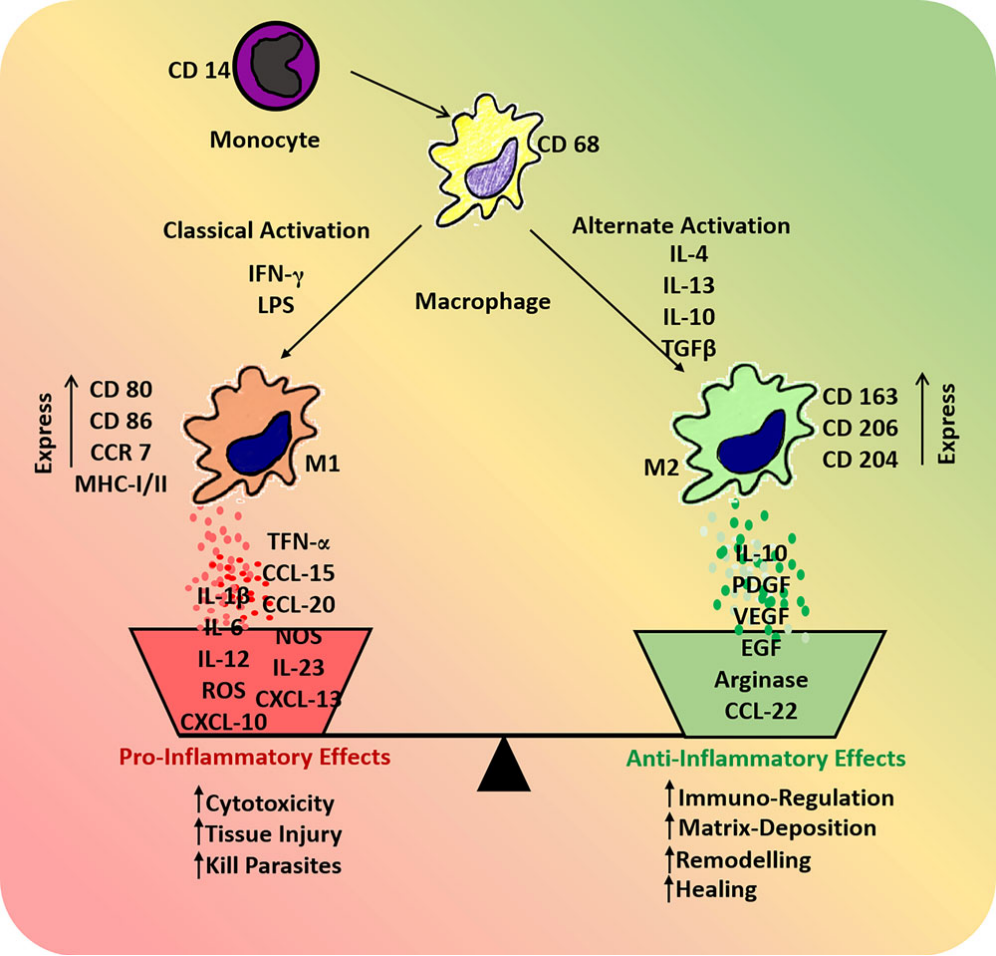
Schematic showing factors involved in macrophage activation and polarization into M1 and M2 subtypes that release specific cytokines and chemokines to determine the type of ensuing inflammatory response. (Online version in colour.)

Regardless of origin, the monocyte–macrophage lineage has considerable plasticity and diversity [39]. They become activated in response to various microbial or environmental signals and differentiate to M1 or M2 phenotypes [40] (figure 1). Classically activated macrophages (M1) emerge following interaction with microbial stimuli, e.g. lipopolysaccharide (LPS) and interferon gamma (IFNγ) [41]. M1 macrophages produce high levels of interleukins, including IL12 and IL23 and inflammatory cytokines IL1β, tumour necrosis factor-α (TNF-α), IL6 and reactive oxygen species (ROS) [42,43]. They act as antigen presenting cells and are involved in TH1 responses, by releasing chemokines including CXCL9, CXCL10 and CXCL13 to attract TH1 lymphocytes [44,45]. M1 macrophages characteristically exert strong anti-microbial and tumoricidal activity [46]. However, alternatively activated macrophages (M2) produce low levels of IL12 and IL23 and high levels of anti-inflammatory cytokines such as IL10 [47]. They characteristically express scavenger, mannose and galactose receptors, which scavenge debris and produce ornithine and polyamines via the arginase pathway [40]. In contrast to M1 macrophages, M2 macrophages do not contribute to antigen presentation and their immunoregulatory properties suppress TH1 inflammatory responses, dampening inflammation through the production of various cytokines and chemokines. These include CCL17, CCL22 and CCL24 to recruit TH2 cells, basophils and mast cells, thereby promoting TH2 responses [40]. They promote angiogenesis and wound healing via the production of platelet-derived growth factor (PDGF), vascular endothelial growth factor (VEGF) and epidermal growth factor (EGF) [45,48].

### 2.3. M1/M2 polarization in regenerative medicine

The M1/M2 macrophage paradigm (figure 1) plays a crucial and dynamic role in the outcome of biomaterial implantation [49]. The initial M1 macrophage response is necessary in the first few days after biomaterial implantation to remove dead cells and tissue debris resulting from surgical incision [50]. M1 macrophages promote angiogenesis [51]. The importance of M1 macrophages in the healing process was shown when exogenous M1 macrophages reduced fibrosis and increased muscle fibre regeneration in a model of skeletal muscle injury [52]. Equally important is the transition of M1 to M2 phenotype for tissue remodelling, as the constant presence of M1 macrophages results in a severe FBR and granuloma formation [49]. While a high M2/M1 ratio may be beneficial for tissue remodelling after biomaterial implantation, the constant presence of M2 macrophages is also problematic as it causes excessive scarring and delays wound healing [32]. Indeed, the proper timing of M1 to M2 polarization and the balance between M1 and M2 numbers determine the success or failure of biomaterial implantation [50]. A precise understanding of this balance is necessary to enhance tissue remodelling and integration of the next generation of biomaterials.

## 3. Foreign body response to biomaterials

**Figure 2.**
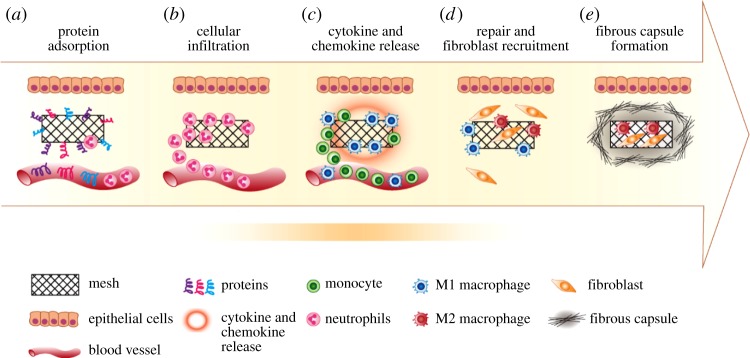
Schematic showing the foreign body response to an implanted inert biomaterial in the host body. (*a*) Protein adsorption; (*b*) cellular infiltration and acute inflammation; (*c*) chronic inflammation, cytokine release and further cell recruitment; (*d*) fibroblast recruitment and collagen matrix deposition; (*e*) formation of fibrous capsule. (Online version in colour.)

An FBR following implantation of a biomaterial is a physiological reaction to a foreign material, a process initiated by protein adsorption and culminating in excessive collagen deposition leading to fibrotic capsule formation around the implant [32]. The physical and chemical properties of the biomaterial, size, topography, chemistry and degradation rate determine the ultimate outcome of the FBR [53]. The FBR commences when the implant contacts the extracellular matrix (ECM), resulting in complement and intrinsic coagulation system activation and immediate blood protein adsorption (albumin, fibronectin, fibrinogen, complement proteins and globulins) on the implant surface. A matrix then forms around the biomaterial [54], prior to interacting with host cells (figure 2*a*). These adsorbed proteins modulate the host cellular response and overall immune response leading to the formation of a provisional matrix, often a thrombus (blood clot) at the interface of the material and host tissue [54]. These proteins, comprising a rich and potent cocktail of cytokines, chemokines, growth factors and cellular secretory components, generate a milieu that attracts inflammatory cells to the implant site (figure 2*b*) [55]. They also provide a structural and biochemical foundation for wound healing processes and modulate the ensuing FBR. The specific proteins that attach depend on the physical and functional nature of the implanted material and the adsorption process is governed by the protein affinity of its surface [56].

### 3.1. Acute and chronic inflammation

As the immune system is triggered, leucocytes, mainly neutrophils and monocytes, rapidly infiltrate the implantation site [32] (figure 2*b,c*). Neutrophils are the primary cellular infiltrators in the initial acute phase. Their emigration from the vasculature into the implant site lasts around 2 days, resulting in their accumulation at the injury site [57]. The neutrophils interact with the biomaterial surface through integrin receptors specific for the adsorbed proteins and a provisional matrix forms, similar to the default process of wound healing (figure 2*c*). The acute phase is also characterized by release of chemo-attractants, histamine and cytokines from mast cell and neutrophil granules, including transforming growth factor *β* (TGFβ), macrophage chemoattractant proteins (MCP1, 2, 3, 4), CCL5 (RANTES), PDGF, CXCL4, leukotriene (LTB4) and IL1β [54,58].

Chronic inflammation follows an unresolved acute phase. The hallmark of chronic inflammation is the presence of mononuclear cells including macrophages and lymphocytes [56]. Their activation leads to further dissemination of chemo-attractants (figure 2*c*). As the macrophages assemble at the site of implantation, they further amplify the chemo-attractive signals through increased production of PDGF, TNF-α, granulocyte colony stimulating factor, granulocyte macrophage colony stimulating factor to recruit more macrophages [32]. Macrophages also play a critical role in wound healing and tissue regeneration. Phagocytosis of wound debris and release of enzymes are important for tissue reorganization. Macrophage release of cytokines and growth factors induces migration and proliferation of fibroblasts (figure 2*d*) and constitute the initial steps toward biomaterial encapsulation and effective tissue regeneration (figure 2*e*) [59].

### 3.2. Foreign body giant cells

**Figure 3.**
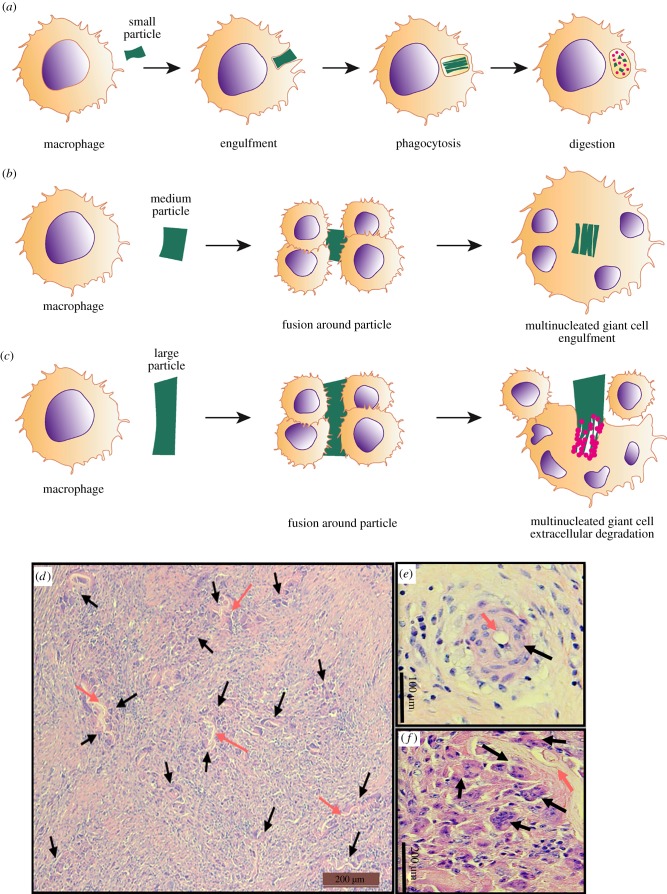
Schematic showing the process of FBGC formation by macrophages responding to foreign particles of different sizes. Macrophages respond to foreign bodies in the host by (*a*) phagocytosis. However, when the particle is larger than a single macrophage, (*b*,*e*) they fuse to form multinucleated FBGCs around the particle, fully encapsulating it. (*c*,*f*) When the particle is much larger than an FBGC, multiple FBGCs attempt to fuse around the foreign particle to render extracellular degradation. (*d*–*f*) Haematoxylin and eosin staining of pig tissue implanted with degradable poly-ɛ-caprolactone nanofibres four weeks after implantation showing the formation of multinucleated FBGCs. (*d*) Low power view showing multiple regions of FBR to the degrading biomaterial. Black arrow: FBGC; pink arrow: degraded biomaterial foreign particle. (Online version in colour.)

Chronic inflammation can progress to a granulation tissue phase, in which the deposition of new ECM and robust angiogenesis into the implantation site are conspicuous. The presence of a non-degradable biomaterial and persistence of granulation tissue eventually results in the formation of foreign body giant cells (FBGCs), where multiple macrophages in contact with each other fuse around the implanted biomaterial (figure 3*c*) [60,61]. The classic histologic description of an FBR consists of macrophages and FBGCs typically located in close proximity of host–biomaterial interface. Macrophages and FBGCs initially attempt to phagocytose and degrade the foreign implanted material. If the FBGCs do not succeed (figure 3*a*), they remain at the biomaterial–tissue interface and shape podosomal structures forming a closed compartment between their surface and the underlying tissue (figure 3*c*,*d*) [58]. This is prominent for implanted non-degradable biomaterials, which are not phagocytosable [51] and can also be observed for degradable biomaterials that are not sufficiently degraded in the early stages to enable complete phagocytosis (figure 3*d–f*) [62]. Although the mechanism of FBGC formation and their exact role in the chronic inflammatory response to biomaterials remain unclear, their presence is traditionally used as a marker of a negative FBR [32] and a major sign in determining the biocompatibility of implants [63]. FBGCs display reduced phagocytic activity but enhanced degradative capacity [55]. Macrophages and FBGCs release matrix metalloproteinases (MMPs) especially MMP-8, MMP-13 and the gelatinases MMP-2 and MMP-9 which may play a pivotal role in biomaterial encapsulation and angiogenesis [60,64]. FBGCs are associated with the release of anti-inflammatory cytokines IL-10 and IL-1RA [63]. Collagen fibres deposited around the implant remodel over time and ultimately contract to form a dense, acellular, fibrous capsule around dense biomaterials that isolates the foreign material from the tissue [65]. Collagen fibres form throughout porous biomaterials such as knitted meshes used for transvaginal implants [51,66]. Fibrous encapsulation marks the remodelling phase of the FBR. By contrast, where no foreign body is present during wound healing, fibroblasts produce collagen to replace the ECM lost during tissue injury without fibrous capsule formation or any particular orientation resulting in healthy, loose connective tissue [67].

### 3.3. Molecular pathways in the foreign body response

Macrophage activation and fusion play an important role in the success or failure of implanted biomaterials. Several molecular mechanisms are activated at each step of the FBR process [32,68,69]. Inflammasome formation during the acute phase of the FBR is one mechanism [70]. The inflammasome is a set of cytosolic proteins including nucleotide-binding domain, leucine-rich repeat-containing type (NLRP), apoptosis-associated speck-like protein containing *CARD* (Asc) and caspase-1 [71]. The interaction of toll-like receptor 4 (TLR4) with NLRP induces pro-IL1β production and activates the inflammasome [72]. The main role of the inflammasome is to convert pro-IL1β to active IL1β for secretion into the extracellular environment. Particles derived from polyethylene-based implants induce the production of pro-IL1β and in turn IL1β release from macrophages [73]. Additional signalling pathways, NF-Κb, JAK/STAT and TNF-α, also play key roles in the FBR [69,74]. Indeed, TNF-α is a key marker of inflammation and FBR where the effect of biomaterial topography or biocompatibility of hydrogels were assessed [75,76].

The JAK/STAT signalling pathway is activated in the FBR when IL-4 binds to its receptor on macrophages, inducing the phosphorylation of STAT6, which translocates to the nucleus and upregulates the expression of E cadherin and β catenin [77]. Upregulation of these adhesion molecules enhances cell–cell interactions and induces the fusion of macrophages [78]. IL-4 also increases signalling through the adaptor protein DAP12, a general macrophage fusion regulator that modulates genes mediating macrophage fusion including *DC-STAMP* (dendritic cell-specific transmembrane protein) [79]. Despite the recognition of several molecular mediators involved in the FBR, the exact molecular mechanisms are still unclear. Control of the FBR to implanted biomaterials necessitates further investigation of other signalling pathways for achieving optimal results.

## 4. Mesenchymal stem cells: mechanisms in immunomodulation and immune cross-talk

MSCs are multipotent and clonogenic, self-renewing progenitor cells, first identified in the bone marrow. MSCs have been isolated from most tissues, including adipose, bone marrow, umbilical cord blood, peripheral blood, endometrium, dental pulp, dermis, amniotic fluid, as well as tumours [29,80–82].

Bone marrow is the most studied source of MSCs in tissue engineering constructs for regenerative medicine purposes. The proliferative, regenerative, paracrine and immunomodulatory properties of bone marrow MSCs have been reported in a large number of studies [83,84]

In recent years, adipose tissue has become an attractive source of MSCs for cell-based therapies and regenerative medicine. Adipose-derived MSCs (ADSCs) can be harvested from an ever increasing number of liposuction procedures. ADSCs have similar properties to bone marrow MSCs but these do not decline with the age of the donor and are an alternative source of MSCs in regenerative medicine [29,85].

Regardless of their origin, MSCs are usually defined by their trophic, paracrine and immunomodulatory functions [86]. These non-stem cell properties appear to have the greatest therapeutic impact, evidenced by the large number of MSC-based clinical trials conducted for several life-threatening inflammatory or immune-related diseases [87]. A large body of medical literature indicates that MSCs repair damaged tissues because they respond to inflammation and migrate to injured sites and influence the microenvironment through the release of molecules involved in reparative processes and tissue regeneration [88]. Biomaterial-based delivery of MSCs may benefit organ and tissue repair through paracrine effects. These properties make MSCs an attractive source of cells for seeding on the engineered biomaterials to influence the FBR following implantation [23,89].

### 4.1. Mechanisms in mesenchymal stem cell and immune cell cross-talk

**Figure 4.**
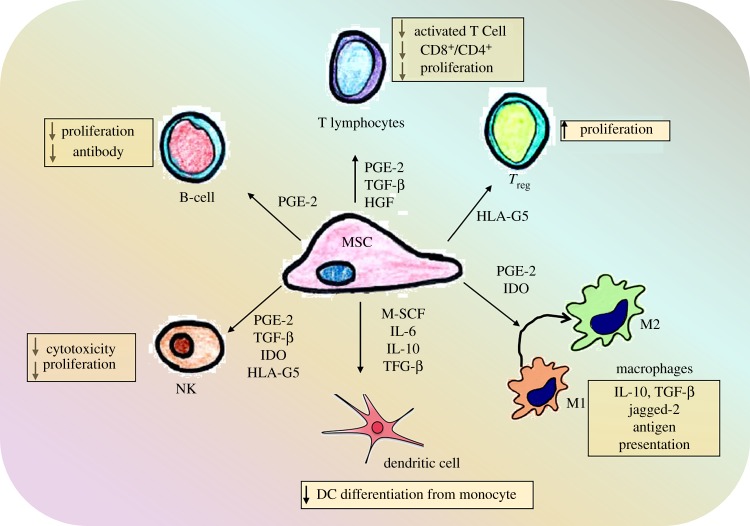
Schematic showing the cross-talk between and its influence on mesenchymal stem cells and cells of innate and adaptive immune system. Adapted from [81,82]. (Online version in colour.)

The immunosuppressive action of MSCs influences the differentiation and function of lymphoid and myeloid cells in a multi-factorial manner [86,90]. Cross-talk between MSCs and immune cells involves several soluble factors released by MSCs (figure 4). In humans, MSCs produce indoleamine 2,3-dioxygenase (IDO) in response to leucocyte IFN-*γ* [91]. In mice, MSCs use an alternative mechanism involving inducible nitric oxide synthase (iNOS) and nitric oxide (NO) [92]. MSCs also mediate T regulatory lymphocytes (Tregs) and T helper-based immunosuppressive activity through the production of heme oxygenase-1 (HO-1) and its metabolic by-product carbon monoxide that mainly impact their recruitment and differentiation [93].

MSCs also produce prostaglandin E2 (PGE2) that has multiple downstream effects including suppression of lymphocyte growth factors (IL-2 or IL-15), differentiation of antigen presenting cells and effector T cells and stimulation of epithelial cell proliferation. MSCs induce macrophages to adopt an enhanced regulatory phenotype via increasing IL-10 and reducing TNF-α and IL-12 secretion predominantly via PGE2 synthesis [94]. MSC-derived soluble factors such as IL-10, PGE2 and IL-1*β* are key molecules involved in the cross-talk between MSC and macrophages, particularly shifting polarization of M1 to the M2 phenotype [95].

Activated T cells influence MSC immunomodulatory properties by secreting pro-inflammatory cytokines IFN-γ and TNF-α which increases the MSC expression of COX2 and IDO, further enhancing macrophage polarization. Macrophage M2 polarization is associated with the induction of Tregs thereby linking to the adaptive immune response [96,97]. In summary, MSCs produce several inducers and mediators that play a role in regulating macrophages that eventually influence all cellular components of the immune system (figure 4). It is also likely that these mediators vary with the local microenvironment and therefore MSC-based therapies involving tissue engineered constructs will likely have varying effects depending on the milieu at the site of implantation.

## 5. Influence of mesenchymal stem cell-based tissue engineered constructs on macrophage responses

The ideal source of MSCs is debated and the varying protocols for their isolation, expansion and ‘stemness’ maintenance has appeared as the biggest challenge in their clinical application. We discovered a small population of clonogenic stromal cells (colony-forming unit fibroblasts) in human endometrium [98] that are highly proliferative, self-renew *in vitro,* differentiate into four mesodermal lineages, osteoblasts, chondrocytes, smooth muscle cells and adipocytes, and expressed typical MSC surface markers (eMSCs) [99]. We also discovered SUSD2 as a single surface marker enriching for clonogenic eMSCs and showed their perivascular identity in human endometrium [100]. We have developed methods for culture expansion of SUSD2^+^ eMSCs in serum-free medium under physiological O_2_ concentrations (5%) [101] using a small molecule TGFβ-receptor inhibitor, A83-01, that maintains MSC stemness [102,103]. A83-01 promotes the proliferation of eMSCs, blocks apoptosis and senescence, maintaining their MSC function. Monitoring these cultures using SUSD2 enables us to produce culture-expanded eMSCs of 85–95% purity after many passages, ideal for quality assurance when using them for autologous or allogeneic clinical applications [29].

eMSCs are easily obtained from endometrial biopsies in an office-based procedure without using an anaesthetic, making them an ideal source of therapeutic cells for pelvic floor tissue engineering [23]. eMSCs can be isolated from regenerated post-menopausal endometrium after women have taken short-term oestrogen. Women can use their own autologous eMSCs for application to pelvic floor disorders and beyond and would opt to do so [104]. The immunomodulatory properties of eMSCs have been partially characterized [105,106], showing similar immunomodulation and cross-talk properties to bone marrow MSCs, reflecting their role in scar-free regeneration of endometrial stroma and vasculature every month following menses [33].

### 5.1. Mesenchymal stem cell immunomodulatory function in animal models of pelvic organ prolapse

Our studies have evaluated the role of eMSCs in modulating FBR to novel pelvic meshes using several pre-clinical animal models of POP, both rodent and ovine.

To date, rodents are the most widely used model for POP development and treatment [107,108] due to their cost-effectiveness, availability of transgenic mouse models and their ease of use. However, rodents have short oestrous cycles and gestations, delivering offspring much earlier in their developmental trajectory with much less damage than for humans. Thus, rodents do not develop spontaneous POP, although induced injury models have been reported for SUI [109,110]. Macaques have been investigated as a large animal POP model. Macaque vagina has a similar structure to that of human and is composed of collagen, elastic fibres and smooth muscle and is oestrogen and progesterone sensitive [111]. Macaque fetuses have a large head to body ratio which is important for modelling of spontaneous POP that occurs in women [112]. This animal model has been also used to study the host response to implanted material. A reduced inflammatory response was reported following the implantation of an ECM graft into the macaque vagina compared to polypropylene mesh [113]. However, MSC-loaded biomaterial has not yet been implanted and studied in the macaque model.

Sheep are a cost-effective alternative, also having a similar vaginal anatomy and size as human. They spontaneously develop acute antepartum POP likely due to prolonged labour and delivering a fetus with a large head [30,107,108]. Detailed physical and histological analysis of ovine vaginal tissue revealed weakening of the vaginal wall with increasing parity in a subpopulation of sheep recapitulating the human condition [114]. In particular, alterations to the ECM composition of the ovine vagina, such as an increased elastic fibre content, possibly a compensatory mechanism to overcome a diminished smooth muscle layer in multiparous sheep, which ultimately may result in the development of POP [30,114].

**Figure 5.**
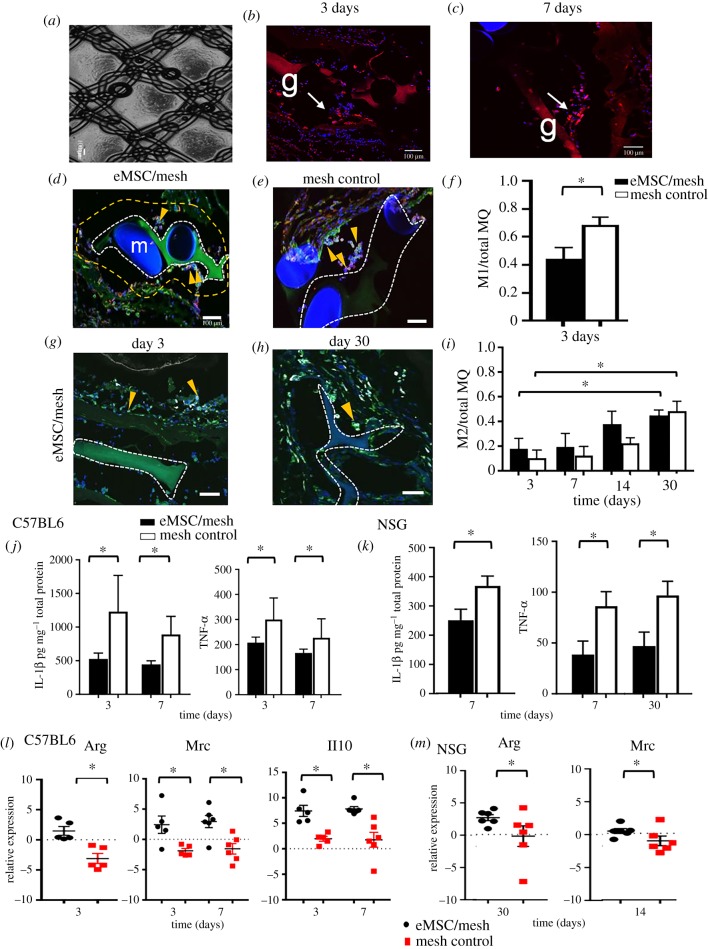
Endometrial MSC transduction and survival on the PA+G mesh in NSG mice. (*a*) Polyamide/gelatin (PA/G) mesh seeded and cultured with mCherry transduced eMSC. (*b*) mCherry labelled eMSC survived 3 and (*c*) 7 days post implantation. Immune response to PA/G mesh seeded with eMSC. (*d*,*e*) CCR7 M1 macrophages (red) co-localized (yellow) with the pan F4/80 macrophage marker (green) around implanted mesh in mesh/eMSC and mesh control groups in C57BL6 mice. (*f*) The ratio of M1 macrophages to total macrophages (MQ) in the first 100 µm increment around mesh filaments 3 days post implantation in C57BL6 mice. (*g,h*) CD206 M2 macrophage (white) co-localized with the pan macrophage F4/80 marker (green) around implanted mesh in mesh/eMSC and mesh control groups in C57BL6 mice. (*i*) The ratio of M2 macrophages to total macrophages (MQ) in the first 100 µm increment around mesh filaments in C57BL6 mice. Inflammatory M1 macrophage cytokine secretion. (*j*,*k*) IL-1*β* and TNF-α secretion in eMSC/mesh and mesh control group implants in (*j*) C57BL6 and (*k*) NSG mice. mRNA expression of M2 macrophage markers. (*l*,*m*) *ArgI, Mrc1 and Il10* in eMSC/mesh and mesh control groups in (*l*) C57BL6 and (*m*) NSG mice. Data are mean ± s.e.m. of *n* = 6 animals/group. *p < 0.05. Adapted from [111]. (Online version in colour.)

Our new alternative non-degradable, lightweight polyamide/gelatin mesh has been purpose designed for POP as it biomechanically matches human vaginal tissue [51,66]. Recently (figure 5), we assessed the immune regulatory effects of eMSCs in immunocompetent (C57BL6) and immunocompromised (NSG) mice implanted with our eMSC/polyamide/gelatin tissue engineering construct (figure 5*a*) [105]. We found that the inclusion of eMSCs in the mesh reduced inflammatory cytokine (IL-1*β*, TNF-α) secretion in NSG and C57BL6 mice (figure 5*j*,*k*). eMSCs also reduced the CCR7^+^ M1 macrophages surrounding the mesh filaments and increased the M2 macrophage marker *Arg*-1, mannose receptor (*Mrc*) and *Il10* mRNA expression within one week following implantation (figure 5*l*,*m*) in C57BL6 mice. We also detected genetically labelled eMSCs surviving for 7 days in NSG (figure 5*b*,*c*) but not C57BL6 mice which resulted in a delayed immunoregulatory effect of eMSCs (30 days) in this immunocompromised mouse model [105]. In a separate study, we found that eMSCs cultured in A83-01 medium maintained their survival following implantation in mice [115]. We further analysed the tissue repair properties of human SUSD2^+^ eMSC/polyamide/gelatin constructs in a subcutaneous wound repair model using immunocompromised nude rats [34]. Despite surviving only two weeks in this xenogeneic model, the eMSCs had a pronounced effect in promoting early neovascularization (one week), likely mediated by the greater influx of M1 macrophages compared to polyamide/gelatin mesh alone [34]. By four weeks, the M1 macrophages had switched to an M2 wound healing phenotype and by 13 weeks, the chronic inflammatory response was reduced with fewer CD68^+^ macrophages surrounding individual polyamide filaments at the tissue interface in comparison to polyamide/gelatin mesh alone. The new collagen fibres deposited around the eMSC/polyamide/gelatin mesh showed physiological crimping without scarring, demonstrating that a cell-based biomaterial implant favours a reduced FBR, improved biomechanical properties of the mesh/tissue complex and better healing in the long term [34,116]. To minimize the FBR using MSC-based meshes, we have developed an ovine model of prolapse [114,117] to enable assessment of vaginally placed eMSCs/polyamide/gelatin tissue engineering constructs for determining site-specific FBR modulation by eMSCs as we translate our findings into the clinic.

Several leading groups in pelvic floor disorders have used MSCs from several sources to modulate the FBR to implanted degradable meshes. Electrospun nanofibre meshes of polylactic acid with adipose-derived MSCs permitted the infiltration of macrophages throughout their entire thickness highlighting their ability to tackle bacterial infection if needed. Additionally, host cell infiltration indicated the desired remodelling of the implant leading to good integration into the host [118]. Amniotic fluid-derived MSCs seeded onto decellularized small intestinal submucosa significantly reduced the expression of the pro-inflammatory cytokine *TNF-α* and *iNos* mRNA at the matrix interface. However, there were no significant differences in anti-inflammatory markers suggesting that the source of MSCs likely influences the FBR [33,119]. Very recently, we showed the potential of endometrium-derived MSCs in improving tissue integration and promoting anti-inflammatory response to degradable nanofibre meshes for pelvic floor disorders. The composition of nanofibre meshes greatly influenced MSC biocompatibility *in vitro* which ultimately impacts implant fate *in vivo* in the long term. Our study showed that biomimetic nanostructured meshes can act as a platform to bring together therapeutic MSCs and host cells and such an interaction can be used to control the FBR favourably in the long term [33].

### 5.2. Mesenchymal stem cell immunomodulatory function beyond pelvic floor disorders

MSC-based biomaterial implants have been widely used in applications beyond pelvic floor disorders and tissue regeneration with similar results of improved neovascularization, M2 macrophage response and reduced fibrosis [26,120]. The mechanism of MSC interaction with inflammatory cells has been investigated in a rat myocardial infarction and reperfusion model using a poly(ethylene)glycol hydrogel to promote repair [121]. Human bone marrow MSCs in alginate hydrogels acted via a CD73-dependent mechanism to increase the bioavailability of adenosine, inhibiting immune cell infiltration and preventing ROS formation [121]. These findings suggest that surface modifications together with MSCs convert pro-inflammatory adenosine monophosphate to anti-inflammatory adenosine, subsequently reducing the FBR. Stromal cell-derived factor 1 (SDF-1) has also been employed in recruiting macrophages to MSC-based implanted biomaterials thereby controlling the FBR [122]. Curcumin-treated MSC sheets improved engraftment *in vivo* and promoted MSC SDF-1 production, facilitating infiltration of M1 macrophages at 7 days that rapidly polarized towards M2 macrophages [123]. Collagen deposition and overall neotissue thickness closely resembled natural tissue. Curcumin promoted MSC proliferation and altered the secretion of the ECM proteins fibronectin and collagens I and III, favourably changing the collagen III/I ratio [123]. Similarly, metal-based surface modifications of biomaterials using magnesium regulated MSC behaviour at the biomaterial–tissue interface and the macrophage-mediated inflammatory response to the degradation products. This suggests that including small quantities of particles into polymeric devices is a valuable strategy to reduce host inflammatory responses [124]. For example, an injectable, instantly solidifying coating material of a unique glucomannan polysaccharide with high affinity for macrophages provided a non-toxic three-dimensional hydrogel construct for delivering MSCs in a murine dorsal subcutaneous pocket model. Effective macrophage activation by the glucomannan coating and their confinement at the tissue–scaffold interface improved osteogenic differentiation and improved scaffold–tissue integration [125]. Other scaffolds with a fibrous topography were examined for their capacity to modulate MSC paracrine effect on macrophages [126]. MSCs on these scaffolds secreted higher levels of anti-inflammatory and pro-angiogenic cytokines resulting in improved therapeutic effects in a skin excisional model [126]. The topography of biomaterials can also influence macrophage polarization (figure 4), which in turn attracts endogenous MSCs to tissue injury sites. MSC–macrophage interactions appear critical for improved tissue repair and the design of biomaterials and tissue engineering constructs can be exploited to promote these interactions. Matrix stiffness influences MSCs fate in high-stiffness hydrogels by direct cell–matrix interaction with macrophages, inducing a pro-inflammatory M1 phenotype and highlighting the need for evaluating novel tissue engineering implants *in vivo* [127]*.* The *N*-acetyl glucosamine content of the natural polysaccharide chitosan alters its topographical structure to induce STAT-1 activation and IP-10 release by U937 macrophages [128]. Chitosan also stimulates anabolic responses in M0 macrophages and M2 but not M1 macrophages resulting in greater IL-10 and IL-1RA release compared to IL1β through pathways independent of the IL-4/STAT-6 signalling axis [128]. These polarized macrophages (figure 1) have a differential capacity to attract human bone marrow-derived MSCs *in vitro*: M0 and M2a macrophages, with or without chitosan stimulation, released soluble factors that attracted MSCs, in contrast to M1 macrophages. A growing body of evidence suggests that MSCs exert an anti-inflammatory response to implantable biomaterials, improve vascularization and promote physiological collagen deposition, thereby reducing the FBR. A greater understanding of the biology of MSCs and their interactions with biomaterials and host immune cells in the response to injury is critical for optimal restoration of tissue structure and function to achieve the best clinical outcomes.

## 6. Considerations in biomaterial design applications to improve foreign body response for women's health

**Figure 6.**
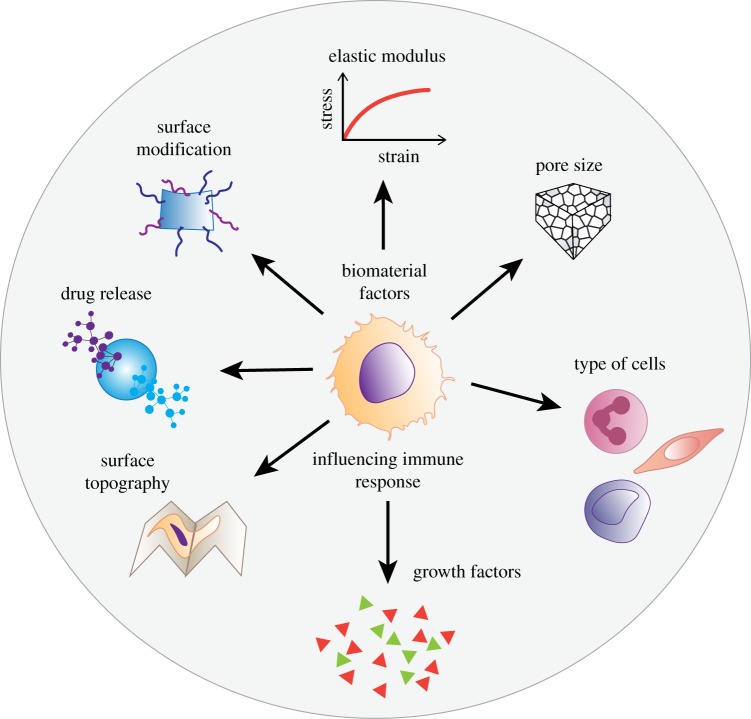
Schematic showing material design factors influencing the macrophage-mediated foreign body response to biomaterial implants including pelvic floor reconstruction. (Online version in colour.)

Recent evidence suggests that promoting specific interactions between cells and the implant can boost immune acceptance and integration of materials with a reduction in the FBR [129]. This has sparked significant interest in developing degradable polymeric meshes including electrospun nanofibre mats and natural decellularized ECM-based meshes [33,130,131]. Irrespective of the choice of material, fabrication strategies to proactively boost the immune system have more benefits than inert meshes (figure 6). The bioengineered immunomodulatory design of materials is likely to modulate the FBR in a more favourable and controlled manner [132]. Design parameters and the ultimate cues presented by biomaterials play a crucial role in modulating the response of host cells [129]. Of these, physical properties such as substrate stiffness, topography, pore size and size of wear debris, and chemical properties including surface chemistry, ligand presentation and release of growth factors can be modified (figure 6) to influence the behaviour of macrophages [49,133]. The plasticity of macrophages furthers the complex interplay between inherent biomaterial properties and those resulting from interactions with the local environment. Pore size modifications of abdominal and vaginal polypropylene meshes have been a major determinant of the FBR [134]. Both small and large pore meshes result in mesh shrinkage; however, it is exacerbated with small pores [135]. As pore size increases, collagen deposition shifts from total encapsulation to improved incorporation with less scar formation and mesh shrinkage; however, over time, large pore knitted meshes also shrink [136]. Early vaginal meshes with extremely small pores hindered macrophage migration, fluid transport and angiogenesis [137]. Newer pre-clinical meshes of polyethylene terephthalate showed that medium to large pore-sized meshes of hexagonal shapes improved tissue ingrowth and peel strength while reducing fibrosis. Similarly, macroporous meshes of polytetrafluoroethylene resulted in better tissue integration compared to small pore sized meshes of the same material [136]. Recently, the performance of a natural ECM bio-scaffold derived from urinary bladder matrix (MatriStem^™^) promoted rebuilding of level I and level II vaginal support without negatively impacting the functional, morphological and biochemical properties of the vagina with minimal FBR [138] in a macaque model.

Inert polypropylene meshes coated with ECM-derived bioactive materials such as cellulose or collagen also favourably influence the FBR. Natural ECM-coated meshes (e.g. collagen) attenuated M1 inflammatory macrophages and the overall FBR by changing the architecture of collagen deposition around the implant [65,139]. Leading groups in tissue engineering for pelvic floor disorders are now turning to biodegradable polymers such as polylactic acid (PLA) and poly-ɛ-caprolactone (PCL). Electrospinning these polymers generates a fine nanostructured topography providing a large surface area to volume ratio, thereby influencing the cellular response in several tissues examined to date. Implanted electrospun nanofibre pelvic meshes of PLA, PCL and even polystyrene show the desired M2 phenotype of murine bone marrow-derived macrophages with production of angiogenic and anti-inflammatory cytokines [140–142]. Biomaterial fabrication methods imparting a nanoscale architecture to scaffolds have a significant positive effect on cell–biomaterial interactions. For example, incorporation of the hormone oestradiol in nanostructured meshes enables higher collagen I, collagen III and elastin production, significantly improving angiogenesis [25,33,143].

Since protein adsorption is the first step in the FBR, early control of the FBR through surface modification is an important consideration in biomaterial design. The delivery of soluble pharmacological anti-inflammatory agents such as dexamethasone, heparin or superoxide dismutase from implant reservoirs and coatings reduces inflammation and fibrous encapsulation [28,144]. Non-degradable polypropylene pelvic meshes have been bioengineered with IL-4 in a layer-by-layer method to overcome FBR limitations, favouring the polarization of macrophages towards an M2 phenotype [145], enabling early anti-inflammatory cross-talk. The gradual release of IL-4 ultimately decreased fibrotic capsule formation, highlighting the importance of early-stage macrophage polarization in optimal biomaterial integration and tissue repair.

Modulation of biomechanical properties such as material stiffness has gained attention in controlling stem cell differentiation *in vitro*. Although the exact effect of stiffness on macrophage phenotype is not fully defined, future investigations will add to the growing body of knowledge demonstrating the influence of biomaterial mechanical properties on macrophage behaviour and the FBR. Investigations suggest that biomaterial topography stimulates changes in macrophage behaviour regardless of biomaterial chemistry. Tailoring the surface chemistry of biomaterials and assessing in MyD88- and TLR-knockout mice demonstrated that antigen presenting cells (DCs) use TLR2, TLR4 and TLR6 to respond to a diverse set of biomaterials [146].

The fibrotic response to biomaterials is multi-factorial with widely varying aetiological and causative mechanisms. Uncontrolled ECM deposition by fibroblasts or myofibroblasts distinguishes fibrosis from controlled tissue repair and is a hallmark of the FBR. This has fuelled significant research into identifying targets, molecules and surface modification methods, to control and coax the immune system into a normal healing pathway. Several factors and molecular pathways are critical to fibrosis development: PDGF, connective tissue growth factor, TGF-β, Notch and Hedgehog signalling pathways [147]. A relaxin receptor (RXFP1) agonist, ML290, is an anti-fibrotic agent with a long half-life and high stability, which has long-term beneficial actions on markers of fibrosis in human cardiac fibroblasts [148]. A peptide agonist of RXFP1 prevented and reversed organ fibrosis and dysfunction in three pre-clinical rodent models of heart or lung disease with similar potency to H2 relaxin [148]. The peptide caused a potent anti-fibrotic effect by modulating fibroblast expression of the pro-fibrotic transcription factor EGR-1, reducing the production of type I collagen and fibronectin, and inhibiting the expression of lysyl oxidase, the main collagen cross-linking enzyme [149]. The use of small molecule anti-fibrotic agents in combination with biomaterials has the potential to improve the FBR and therefore reduce implant-related fibrosis (figure 6). Future studies aimed at understanding the mechanism of action of such agents in combination with biomaterials will shed light on the molecular mechanisms involved in the FBR [150] and could be exploited for improving biomaterial implants used for treating pelvic floor disorders.

## 7. Conclusion

The FBR is a major obstacle towards long-term functionality of materials and devices implanted in the body including for pelvic floor reconstruction. Typically, implanted biomaterials trigger an FBR almost immediately, followed by a dynamic inflammatory process that involves cross-talk between multiple cell types. The FBR is not a single response, but a cascade of interlocked events. Contrary to traditional approaches, current strategies to improve the FBR involve immunomodulatory tissue engineering approaches combining therapeutic MSCs together with surface modifications and other aspects of material design. Such approaches aim at initiating a proactive immune response at an early stage to ensure control of biomaterial fate. Lessons from the complications arising from pelvic meshes and ongoing research to suppress the deleterious FBR over the next decade will see the development of new constructs that will soon be tested in clinical settings. Further elucidation of the FBR and development of novel biomaterials and new methods to suppress it will likely continue as a focus of research.

## Data accessibility

This article has no additional data.
